# Supplementary material for: Mask adherence and rate of COVID-19 across the United States
Source: PLoS One. 2021 Apr 14;16(4):e0249891. doi: 10.1371/journal.pone.0249891 (PMC8046247; doi:10.1371/journal.pone.0249891)
Supplement: Attachment — Submitted filename: PLOS_one_MS review.docx [file pone.0249891.s002.docx]

MS review PONE-D-21-01163

Mask wearing has been advocated by public officials as an important way to decrease the spread of COVID-19. Nevertheless, mask-wearing policies and adherence have varied considerably among the states over the course of the pandemic. This study analyzes publicly available data from all states from April 1 through Oct 31 to estimate the effect of mask wearing on COVID-19 rates during the summer and early fall of 2020. Research on public health policies and lack of mask wearing is warranted to estimate the myriad of downstream effects.

Strengths:

1. This manuscript includes publicly available data from all 50 states and DC on demographics, mask-wearing and physical-distance policies, mask-wearing adherence, and COVID-19 cases. Therefore, no additional data collection was necessary.
2. Data on mask use was obtained from the IHME website, which comprises information from “Premise, Facebook Global Symptom Survey (research based on survey results from the University of Maryland Social Data Science Center), Kaiser Family Foundation (KFF), and YouGov COVID-19 Behaviour Tracker survey.”
3. The analyses take into consideration the delayed effect of mask-wearing policies and adherence on COVID-19 health outcomes.
4. It is useful that the authors opted to present individual state data so that state leaders can potentially incorporate the findings into their future public health policymaking and messaging.

Queries:

1. Table 1 broadly categorizes the states and DC according to their demographics, mask-wearing policies, mask-wearing adherence, and COVID-19 cases. However, this table inaccurately lists WA and OR as among the states with no mask policy from April-August. WA implemented mandatory masking policies on June 26, 2020, and July 24, 2020, and at least one of these orders met the authors’ criteria as a “strict” policy. Likewise, OR implemented initial mandatory masking policies on July 1, 2020. We have not checked other state categorizations in this table, but these errors suggest the possibility that other errors may exist in the data and that the analyses in the manuscript may be flawed.
2. IHME projects that 95% mask use reduces COVID-19 case rate by 30% or more, whereas the observed rate of mask use in August and September 2020 nationally was approximately 65%. Following IHME’s projections, that suggests an anticipated reduction in COVID-19 rate that would be less than the above estimated 30%. Given that this study analyzed mask use and COVID-19 rate for each state, it would be useful if the authors provided and clearly stated COVID-19 rate reductions, even though they may vary by state.
3. The authors need to carefully compare the description of their results to their methods and to the depiction of their results in Figures 1 and 2, as they do not seem to match.

First, in the text they state that 16, 18, 16, and 30 states had high rates of adherence in the months of June, July, August, and September, respectively, whereas Figure 1 seems to show that only 13 states had high rates of adherence for these months. It seems likely that the text description reflects an inconsistent or confusing application of their quartile method of categorizing mask adherence—indeed, isn’t it nonsensical to describe 16, 18, or 30 states as having high adherence given their previous description of high adherence as belonging to the upper quartile, which would presumably consist of 13 states? It may be useful for the authors to either reconsider this quartile method or to offer more insight into this analytic method elsewhere in the paper and to check that it is consistently applied to all mentions of mask-wearing adherence.

Second, the sentence about Arizona, Connecticut, Hawaii, Massachusetts, Michigan, New York, Rhode Island, Vermont, and DC is unclear; it should presumably begin by stating that 9 of the 13 states/DC in the high-adherence quadrant did not experience a high COVID-19 rate in the subsequent month.

Third, the text related to Figure 2 speaks of “no” mask wearing policy and “any” mask wearing policy, whereas Figure 2 itself depicts “no” mask wearing policy and “some” mask wearing policy. Given the description in the Methods section, the Figure 2 language of “some” presumably would include both “recommended” and “strict” mask wearing policies, though this is certainly not spelled out. Moreover, the percentages mentioned in the text, do not necessarily seem to match the percentages depicted in the figure. For instance, the text indicates that 40 to 73% of no policy states had high infection rates, whereas the figure visually seems to show 50 to 73%. Likewise, the text indicates that less than 20% of states with a mask-wearing policy had high COVID-19 rates in the first three months studied, whereas the figure seems to depict about 25% for these states for July/August and August/September.

4. It may be useful for the authors to comment in the discussion section on the notable spike seen in October for COVID-19 infections among states that had some mask-wearing policies in place in September. That is, do the authors draw any implications from the data or from other factors for the potential decrease in the efficacy of masking behaviors/policy compared to non-masking behaviors/policy for that month?
